# Gut microbial metabolism in Alzheimer's disease and related dementias

**DOI:** 10.1016/j.neurot.2024.e00470

**Published:** 2024-10-28

**Authors:** Jea Woo Kang, Vaibhav Vemuganti, Jessamine F. Kuehn, Tyler K. Ulland, Federico E. Rey, Barbara B. Bendlin

**Affiliations:** a Wisconsin Alzheimer's Disease Research Center, University of Wisconsin School of Medicine and Public Health, Madison, WI, USA; b Department of Bacteriology, University of Wisconsin-Madison, Madison, WI, USA; c Department of Pathology and Laboratory Medicine, University of Wisconsin-Madison, Madison, WI, USA; d Wisconsin Alzheimer's Institute, School of Medicine and Public Health, University of Wisconsin-Madison, Madison, WI, USA

**Keywords:** Alzheimer's disease, Dementia, Gut microbiome, Metabolites

## Abstract

Multiple studies over the last decade have established that Alzheimer's disease and related dementias (ADRD) are associated with changes in the gut microbiome. These alterations in organismal composition result in changes in the abundances of functions encoded by the microbial community, including metabolic capabilities, which likely impact host disease mechanisms. Gut microbes access dietary components and other molecules made by the host and produce metabolites that can enter circulation and cross the blood-brain barrier (BBB). In recent years, several microbial metabolites have been associated with or have been shown to influence host pathways relevant to ADRD pathology. These include short chain fatty acids, secondary bile acids, tryptophan derivatives (such as kynurenine, serotonin, tryptamine, and indoles), and trimethylamine/trimethylamine N-oxide. Notably, some of these metabolites cross the BBB and can have various effects on the brain, including modulating the release of neurotransmitters and neuronal function, inducing oxidative stress and inflammation, and impacting synaptic function. Microbial metabolites can also impact the central nervous system through immune, enteroendocrine, and enteric nervous system pathways, these perturbations in turn impact the gut barrier function and peripheral immune responses, as well as the BBB integrity, neuronal homeostasis and neurogenesis, and glial cell maturation and activation. This review examines the evidence supporting the notion that ADRD is influenced by gut microbiota and its metabolites. The potential therapeutic advantages of microbial metabolites for preventing and treating ADRD are also discussed, highlighting their potential role in developing new treatments.

## Graphical abstract

**Figure:**
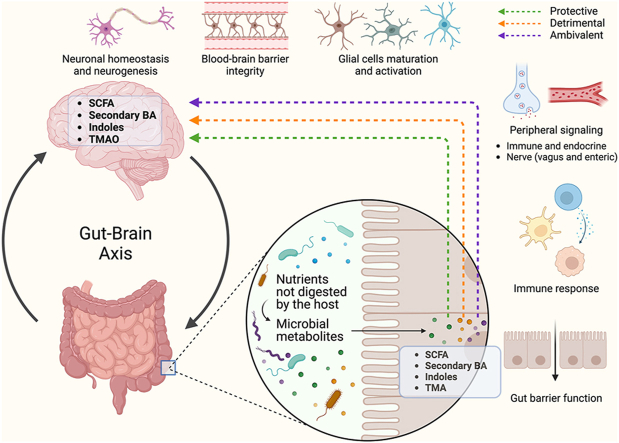

## Introduction

Alzheimer's disease (AD) is a progressive neurodegenerative disease affecting approximately 6.9 million Americans aged 65 and older, and approximately 50 million people worldwide are living with Alzheimer's disease and related dementias (ADRD). According to the Centers for Disease Control and Prevention (CDC), AD is the seventh-leading cause of death in the United States (US). While AD is the most common cause of dementia, other forms of dementia considered under the umbrella of ADRD are Lewy body dementia, frontotemporal dementia, vascular dementia, and mixed (multiple-etiology) dementia [1].

There are no current treatments that cure or reverse AD; however, the US Food and Drug Administration (FDA) has approved treatments under two categories: disease-modifying drugs that impact the pathology of AD (specifically amyloid), and drugs that may temporarily mitigate some symptoms of AD. The FDA-approved drugs Lecanemab (Leqembi®) and Donanemab (Kisunla®) modify disease progression by removing β-amyloid plaque (amyloid β; Aβ), which is a defining feature of AD [2] and attenuate cognitive and functional decline in AD [3,4]. Other drugs approved to treat symptoms of AD include cholinesterase inhibitors (donepezil, rivastigmine, and galantamine) and glutamate regulators (memantine) [5,6]. Other drugs and interventions are used to manage behavioral and psychiatric symptoms that accompany dementia [7,8]. While therapeutic options for ADRD are limited, clinical trials are ongoing aimed at multiple targets including Aβ, tau, vascular, immune function and inflammation, metabolism, synaptic plasticity, and neuroprotective mechanisms [9].

Intervening to lower the risk of developing ADRD may be facilitated by early disease detection. Biomarkers, including fluid [blood (plasma) and cerebrospinal fluid (CSF)] and imaging (positron emission tomography; PET) based measures, have significantly advanced early detection of ADRD pathology. In the context of AD, the A/T/N system – A: Aβ/amyloid-based markers, T: tau/neurofibrillary pathology markers, and N: neurodegenerative or neuronal injury markers – provide a framework for detecting and staging AD [10,11]. Due to the advancements in biomarker development, it has become possible to detect AD more readily in living people and intervene to slow disease progression. The use of *in vivo* biomarkers can also inform on the mechanisms by which risk factors for dementia impact the brain and ADRD pathology. Several potentially modifiable risk factors for dementia have been identified, many of which impact, or have been associated with, gut microbiome alteration, including vascular risk factors, smoking, obesity, depression, physical inactivity, diabetes, social isolation, alcohol consumption, and air pollution [12].

The adult mammalian gut is colonized by microbes that encompass the three domains of life, including bacteria, archaea, and eukarya, and a vastly diverse pool of viruses and obelisks (viroid-like elements). Intestinal bacteria have been extensively studied in the context of host health and the field uses the term “gut microbiome” even when only referring to the bacterial component of the gut. We will use the term in a similar fashion. The gut microbiome complements host biology by providing essential functions such as the breakdown and fermentation of dietary components that reach the distal intestine, modulation of intestinal epithelial energy homeostasis, barrier function, immune system regulation, and resistance to pathogen colonization [13,14]. The effects of the gut microbiome extend to other organs including the brain, and the gut and brain are connected via multiple signaling pathways [15]. Gut microbes play an important role in the modulation of these signaling pathways that conform to the gut-brain axis, which may impact not only the intestine but also brain function and neuropathology. Gut microbiome alterations have been consistently documented in AD, and these alterations are also associated with AD pathology as determined by PET and fluid biomarkers of AD [16,17]. Changes in the composition of gut microbes can also lead to alterations in their function. Multiple lines of evidence suggest that microbial metabolism, along with the diverse array of molecules produced and modulated by these microbes, are key mechanisms linking gut microbiota to neurodegenerative diseases [[18], [19], [20], [21], [22]].

**Fig. 1:**
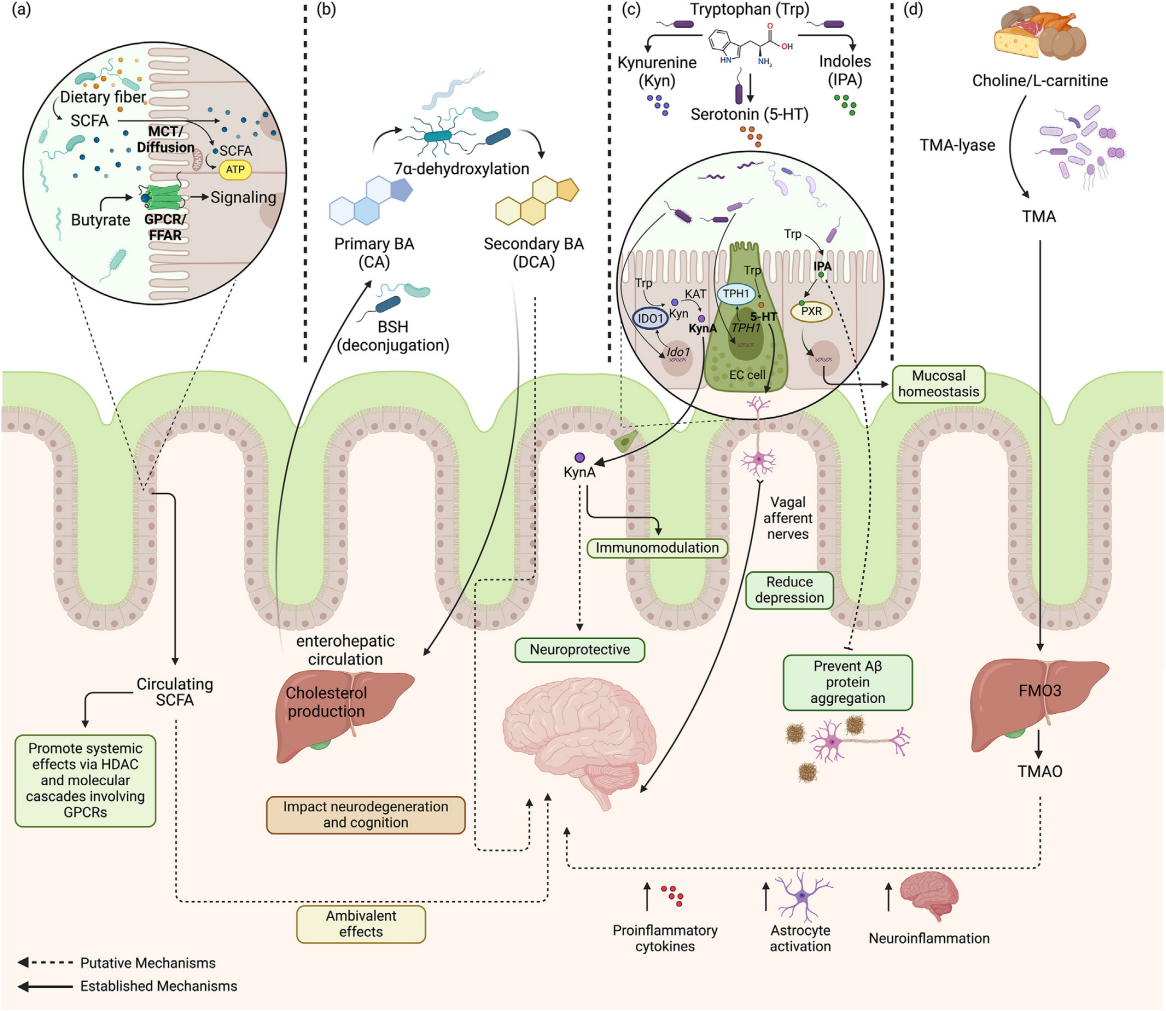
**Microbial metabolites and their potential role in ADRD pathology.** (a) Short chain fatty acids (SCFA) are synthesized during bacterial fermentation, mostly from carbohydrates that reach the distal gut (e.g., dietary fibers), SCFA—particularly butyrate—are an important source of energy for colonocytes. SCFA effects also include epigenetic modulation and activation of molecular cascades involving G-protein coupled receptors (GPCRs) [23]. SCFA act locally, but there is also evidence that they enter systemic circulation. SCFA have been associated with both positive and negative impacts on ADRD [24,25]. (b) Primary bile acids (BAs) are conjugated with glycine or taurine to form conjugated primary BAs which are among the bile salts in the liver. Bile is stored in the gallbladder and released into the duodenum to emulsify fats for lipid digestion and absorption. Conjugated primary BAs are deconjugated by microbial bile salt hydrolases (BSH) and converted to secondary BAs [26]. Previous work suggested that secondary BAs are associated with A/T/N biomarkers for AD, cognitive decline, and anxiety and depression [[27], [28], [29]]. (c) Gut microbes are able to regulate production or directly synthesize tryptophan (Trp) derivatives. Gut microbes can modulate indoleamine-2,3-dioxygenase 1 (IDO1) expression level in enterocytes which converts Trp to kynurenine (Kyn) [30,31]. Kyn can be further metabolized by kynurenine aminotransferase (KAT) to kynurenic acid (KynA) which is reported to exert immunomodulatory activities and potential neuroprotective effects [32,33]. Serotonin (5-HT) is a neurotransmitter produced in enterochromaffin (EC) cells from tryptophan catalyzed by tryptophan hydroxylase 1 (TPH1). The expression level of TPH1 can be regulated by gut microbes [34,35]. 5-HT stimulates neuronal signals to the vagal afferent nerves that are connected to the brain, which may have a potential impact on depression [[36], [37], [38]]. Indole propionic acid (IPA) is one of the indole derivatives produced by bacterial transformation of tryptophan. IPA can act as a ligand for the pregnane X receptor (PXR) and its activation increases the expression of junctional protein in the intestine to promote intestinal homeostasis [39,40]. IPA can cross the blood-brain barrier (BBB) and is suggested to prevent amyloid beta (Aβ) protein aggregation [[41], [42], [43]]. (d) Gut bacterial metabolism of choline or l-carnitine via trimethylamine-lyase (TMA-lyase) produces trimethylamine (TMA) [[44], [45], [46], [47], [48]]. TMA is absorbed into the portal circulation and transported to the liver, where flavin monooxygenases, particularly flavin monooxygenase 3 (FMO3), metabolize TMA to produce TMAO [44,45,49]. TMAO then enters circulation and has a variety of effects including modulation of cholesterol and bile acid metabolism [50], impairment of insulin signaling [51], increased inflammation [52,53], and induction of endothelial and neuronal senescence [54,55]. TMAO is able to cross the BBB and stimulate increased levels of proinflammatory cytokines and markers of astrocyte activation, impaired memory and learning, and increased neuroinflammation [56]. Human cohort studies found that CSF TMAO levels are correlated with AD biomarkers [57], and animal studies have found that in AD mice, TMAO drives phenotypes associated with AD [[58], [59], [60]].

In this review, gut microbial metabolites are considered candidate modifiable factors in the pathophysiology of ADRD that may prevent or exacerbate disease onset and progression. Potential pathology that links gut microbiome with aging and ADRD is also considered. This article further summarizes the impact of gut microbial metabolites highlighting short chain fatty acids (SCFA), bile acids (BAs), tryptophan derivatives, and trimethylamine (TMA)/trimethylamine N-oxide (TMAO) on ADRD pathophysiology and discusses potential therapeutic targets for ADRD by the modulation of these gut microbial metabolites for ADRD prevention and treatment (Fig. 1).

## Gut Microbiome and Age

Complex and multifaceted processes including biological, behavioral, and environmental factors impact biological age. Increasing evidence shows that the gut microbiota composition and diversity changes across the lifespan, and is accompanied in many cases by progressive deterioration in the intestinal tract of the host [[61], [62], [63], [64]]. These changes are likely associated with profound changes in gut microbial functions. Understanding microbial function is crucial to understanding the physiological interactions between microbes and humans both in the context of aging, and more broadly in human health and disease.

Aging is accompanied by changes in homeostasis and metabolic functions of the host and also by alterations in the microbiome. Changes in the microbiome may in turn influence the rate of age-related physical and cognitive decline [64]. For example, several studies suggest that the taxa responsible for breaking down dietary fiber, decrease with age, while the organisms with potential for causing disease increase with age [[65], [66], [67]]. While the gut microbiome composition is largely stable throughout most of adult life, it remains largely unknown at which point in the lifespan the gut microbiome composition shifts to reflect a more “aged” state, as the composition gradually changes over time [68]. Studies have reported that among older individuals, the phylum Bacteroidota (Bacteroidetes) was more predominant than Bacillota (Firmicutes) [69,70]. Conversely, Firmicutes to Bacteroidetes ratio (F/B ratio) showed a tendency to increase with age [71]. Moreover, factors such as diet, and cultural differences may be responsible for differences in gut microbiome composition across age. Additionally, it's important to consider changes in gut microbes at lower taxonomic levels and among less prevalent taxa. A reduction in the relative proportion of microbes such as bifidobacteria or lactobacilli and an increased abundance of *Akkermansia* has been linked to characteristics of the microbiome in older individuals or patients [16,62,63,[72], [73], [74], [75]]. Gut microbiome functions, including those related to carbohydrate metabolism, amino acid biosynthesis, and butyrate production are also decreased in older adults [62].

Aging may lead to structural and functional changes in the gut microbiome, affecting the production of diverse microbial metabolites such as SCFA [76], TMAO [55], tryptophan derivatives [77], and several others [64]. Gut microbes and the host interact in diverse and complex ways, where metabolites represent a major node of communication between microbes and the host. These microbial metabolites locally and systemically influence physiology and are suggested to affect age-associated inflammation (inflammaging) [78,79], age-related cognitive decline and dementia [80], and age-dependent changes in oxidative stress [81,82] in both animal and human studies.

A study showed that the F/B ratio was shifted after middle cerebral artery occlusion (MCAO) in aged and young mice [83]. The F/B ratio increased, similarly to MCAO, regardless of the age of the recipient mice when the aged donor microbiome was transplanted. In addition, increased mortality and cytokine levels and decreased performance in behavioral testing were observed in young mice exposed to MCAO after being transplanted with the microbiome of aged mice. Increased survival and improved recovery were observed in aged mice that underwent MCAO after being transplanted with the microbiome of young mice [83]. They also showed that aged stroke mice who received fecal transplants from young mice exhibited less behavioral impairment and reductions in brain and gut inflammation compared to the mice that received fecal transplants from aged mice [84]. Moreover, transplantation of selected bacterial strains from the young mice to aged mice with stroke resulted in the mitigation of post-stroke neurological deficits and inflammation, and increased levels of gut, brain, and plasma SCFA [84]. These studies suggest that gut microbiome modification may impact age-related diseases, which may be mediated by bacterially produced metabolites. In C57BL/6 ​J mice, gut bacterial taxa such as *Alistipes* were more abundant within the old (857 ​± ​16 days-old) and middle-aged (589 ​± ​18 days-old) mouse groups [85]. Functional analysis revealed that functions related to fructooligosaccharides and raffinose utilization and lactate utilization were lowered in old mice compared to the middle group. Furthermore, functions including cobalamin (vitamin B12) and biotin biosynthesis and bacterial genes (*uvrABC* and *uvrD*) related to DNA repair were underrepresented in older mice [85]. In a clinical study, shifts in serum and fecal metabolites as well as fecal microbiome were observed in older adults with frailty compared to middle-aged adults and non-frail older subjects [86]. In particular, the relative abundance of *Roseburia* and *Faecalibacterium* decreased whereas the abundance of *Bacteroides* and *Parabacteroides* increased in frailty older adults. The increased abundance of *Bacteroides* and *Parabacteroides* was associated with lowered fecal levels of dodecanedioic acid and serum levels of indole [86]. Thus, accumulating evidence suggests that aging alters the composition and function of the gut microbiome as well as the microbial and host metabolome, potentially impacting the host's health and disease. However, these changes can be prevented and/or possibly reversed by endogenous and exogenous factors that influence the gut microbiome.

Environmental factors, most prominently diet, influence gut microbiome composition. One diet that has been extensively explored in the context of aging is the Mediterranean diet, which comprises a high intake of vegetables, legumes, fruits, nuts, olive oil, and fish and a low intake of red meat, dairy products, and saturated fats. One study, the NU-AGE dietary intervention study, examined the effect of the Mediterranean diet on more than 1200 elderly individuals for 12 months across five European countries (Poland, Netherlands, United Kingdom, France, and Italy) [87]. This study showed that more strict adherence to a Mediterranean diet significantly improved global cognition and episodic memory compared to those adults with lower adherence [88]. Microbiome analysis in a subset of participants revealed that adherence to this diet shifted gut microbiome composition and positively correlated with reduced markers of frailty and inflammation as well as improved cognitive function [89]. In addition, changes in microbial metabolites including increased production of short/branched chain fatty acids (SCFA/BCFA) and lower production of secondary bile acids, p-cresols, ethanol, and carbon dioxide were observed [89].

Changes in host physiology related to aging may also influence the gut microbiome. Age-related alterations in gastrointestinal motility and function such as impaired esophageal peristalsis, gastric emptying, colon transit times, and digestion may lead to lowered food consumption, malabsorption, and malnutrition [90]. Although mechanisms remain unclear, age-related perturbation of intestinal motility has been suggested to drive gut microbial dysbiosis and intestinal permeability, which increases bacterial translocation, infection rates, and inflammation [91,92]. A clinical study showed pronounced differences in postprandial bile acid profiles of young and elderly participants who consumed a liquid mixed meal (16 ​% protein, 35 ​% fat, and 49 ​% carbohydrates) [93]. Microbiome analysis in these individuals showed that aging is associated with increased alpha diversity and a higher relative abundance of *Ruminiclostridium*, *Marvinbryantia*, and *Catenibacterium*. In addition, the elderly showed higher total cholesterol levels across all time points and overall lower appetite and energy expenditure compared to the young participants [93].

Mounting evidence suggests that gut microbiome composition and function are altered with aging. It remains to be elucidated whether age-associated changes in host physiology drive alterations in the gut microbiome, whether modifications in the gut microbiome precipitate age-related physiological changes, or if these processes occur concurrently. More research is needed to understand the underlying mechanisms by which gut microbiome impacts the host physiology in terms of aging.

## Gut Microbiome and Alzheimer's Disease and Related Dementias (ADRD)

### Gut microbiome alterations and their relevance to ADRD pathology

Dysbiosis, defined as an imbalance in the composition and metabolic capacity of the microbiota, is commonly found in many diseases [[94], [95], [96]] (e.g., gastrointestinal diseases) and also in neuropsychiatric and/or neurodegenerative disorders such as AD [63,97], Parkinson's disease (PD) [98,99], schizophrenia [100,101], and autism spectrum disorder (ASD) [102,103]. Dysbiosis in the gut may increase the susceptibility of the intestinal environment to pathogens such as *Clostridium difficile* [104]. *C. difficile* is known to cause diarrhea, inflammation in the gut, and tissue necrosis [105]. Pathogenic infections such as *C. difficile* infection (CDI), which causes local and systemic inflammation, could result in increased intestinal and BBB permeability [[106], [107], [108], [109]]. Increased BBB permeability is detrimental as it exacerbates neuroinflammation which allows for the leakage of water and blood components and metabolites into the brain [[110], [111], [112]]. Therefore, evidence shows that there is a pathological connection between gut microbiome and ADRD.

Several studies have shown that changes in gut microbiome can also lead to improvement in the host brain health. Multiple studies have shown that fecal microbiota transplant (FMT) are effective for treating recurrent CDIs [113]. Two separate FMT case studies reported improvements in cognitive function, mood, and more interactive and expressive affection, as well as resolved CDI following FMT treatment in a 90-year-old woman and an 82-year-old man with CDI who had experienced a gradual decline in memory and cognition [114,115]. Preclinical studies of AD also support this notion: FMT treatment of APPswe/PS1dE9 transgenic mice showed improvement in AD-like pathology (decreased phosphorylation of tau protein and the levels of Aβ_40_ and Aβ_42_) [116], whereas FMT treatment in 5xFAD mice reduced amyloid plaque burden in the brain and improved cognition [117]. Additionally, a study in animals in which fecal microbiota from AD patients were inoculated into young adult rats resulted in memory impairments and decreased hippocampal neurogenesis [118]. Furthermore, FMT from 3 ​× ​Tg-AD (FMT-AD) compared to FMT from young wild-type (FMT-young) mice to young C57BL/6 wild-type mice after controlled cortical impact injury induced larger lesions, increased activated microglia/macrophages, and reduced motor recovery after traumatic brain injury [119]. Taken together, these studies support the notion that gut microbiome alterations can impact ADRD pathology and clinical phenotypes. However, further in-depth research on the pathophysiological mechanisms of microbial functions and associated metabolites is needed, and larger human studies are necessary.

### Gut microbiome-immune interactions and their relevance to ADRD pathology

The gut microbiome is in close proximity to and interacts with the gut immune system. Studies of germ-free models have demonstrated that the presence of commensal or absence of certain bacteria is crucial in immune system development and in maintaining the homeostasis of intestinal immunity [[120], [121], [122], [123]]. For example, in germ-free animals compared to specific-pathogen-free animals, there was an undeveloped mucosal immune system which was characterized by hypoplastic Peyer's patches with few germinal centers and reduced numbers of plasma cells that produce IgA as well as CD4^+^ T cells from the lamina propria [124]. Several human commensal bacteria including *Clostridium* species and *Bacteroides fragilis* also influence the differentiation of regulatory T (T_reg_) cells and the production of interleukin (IL)-10 [[125], [126], [127]]. Finally, a dramatic reduction in Gram-positive bacteria via vancomycin treatment in mice modified the colonic environment to induce the recruitment of monocytes and macrophages [128].

Immunosenescence which is a progressive decline in innate and adaptive immunity is associated with aging and accompanied by inflammaging. Several studies have suggested inflammatory pathways are also altered during aging and aging-associated neuropathology, specifically in AD [125,129,130]. Perturbation of gut microbes also plays a role in intestinal inflammation which can potentially contribute to the pathogenesis and progression of AD. Local infection in the gut increased the production of proinflammatory mediators and the infiltration of immune cells to the infection site in a triple transgenic Alzheimer's disease (3xTg-AD) mouse model, leading to an increased number of activated microglia in the mice and exacerbating neuroinflammation in AD [131]. In a human study, participants with cognitive impairment and amyloid positivity showed a higher expression of proinflammatory genes and lower levels of the transcript for the anti-inflammatory cytokine IL-10 in their blood compared to the control group. The transcript levels of several proinflammatory cytokines in blood from participants correlated positively with the abundance of *Escherichia/Shigella* [132].

Gut microbially-derived metabolites interact with the host immune system. SCFA, for instance, can signal intestinal epithelial cells and immune cells to support intestinal mucosal barrier integrity and immune functions. SCFA affect gut immunity by regulating the differentiation, recruitment, and activation of immune cells including monocytes, neutrophils, dendritic cells, macrophages, and T cells [133,134]. In particular, butyrate facilitates the differentiation of naive CD4^+^ T cells into T_reg_ cells. Butyrate can also bind to G-protein coupled receptor (GPR)109A expressed in epithelial cells or immune cells regulating the production of cytokines such as IL-10, IL-18, and transforming growth factor beta (TGF-β) can trigger other immune cells to sustain homeostasis of the gut immunity [135]. SCFA may prevent the activation of immune cells that may otherwise chronically increase levels of systemic inflammation leading to structural damage to the BBB and enhanced proinflammatory phenotypic changes of glial cells including microglia and astrocytes [[136], [137], [138]]. In late-stage AD where Aβ plaques are accumulated and microglia are in a dystrophic state, microglia may have a compromised phagocytic activity, and the phagocytosis of Aβ species by the microglia may eventually lead to the increased expression of proinflammatory cytokines as well as the release of apoptosis-associated speck-like protein containing a C-terminal caspase recruitment domain (ASC) specks to further promote Aβ aggregation [139].

Taken together, the composition of gut microbes and their metabolites may be crucial modulators of both local and systemic immunity. The gut microbiome may be one of the mediators that exacerbate inflammation-mediated neurodegeneration and Aβ aggregation in AD; however, further examination of mechanisms is needed to understand the precise pathways through which microbial changes influence neuroinflammatory processes and contribute to neuronal damage and Aβ plaque formation.

### Gut microbiome-vagal pathways and their relevance to ADRD pathology

Vagus nerve is a nerve that travels from the brainstem to the gastrointestinal system. The vagal afferent comprises 80–90 ​% of the vagus nerve that signals sensory information from organs to the brain affecting emotions, appetite, and the central immune system [140]. The vagal efferent comprises 10–20 ​% of the vagus nerve that sends signals from the brain to other organs affecting digestive enzyme secretion, peripheral immune system, and energy metabolism [140]. The vagus nerves do not cross the epithelial layer of the gut and, thus, are not in direct contact with gut microbes [141]. However, the majority of the cells such as intestinal epithelial cells, enterochromaffin cells, enteric neurons, and intestinal immune cells communicate with the brain via neural and hormonal pathways which are also impacted by the gut microbiome. Although evidence shows that changes in the composition of gut microbiota could modulate brain function [[142], [143], [144]], it is more likely that the vagal pathway communicates via signaling pathways that involve changes in microbially-derived metabolites. SCFA such as acetate, propionate, and butyrate were shown to suppress food intake in fasted mice, this effect was attenuated by the hepatic vagotomy [145]. It is possible that SCFA may be sending signals to the brain via the vagal afferent pathway and nodose ganglion neurons, which are known to express GPR41, to suppress appetite [141,146,147]; however, more research is needed to fully understand the mechanisms involved and confirm the role of this pathway in appetite regulation. SCFA can also activate enteroendocrine cells via GPR41 and GPR43, leading to the release of hormones such as peptide YY and glucagon-like peptide 1, which regulate appetite, metabolism, and gut motility [[148], [149], [150]].

Several studies have examined the mediating role of the vagus nerve in AD. A CCAAT-enhancer-binding protein (C/EBPβ) is an effector that accelerated cognitive dysfunctions in young 3xTg mice via increasing delta-secretase (δ-secretase) expression [151]. Recently, C/EBPβ/δ-secretase was shown to be activated in an age-dependent manner in the colons of 3xTg mice and AD patients, initiating the formation of Aβ and Tau fibrils that spread to the brain [152]. Treatment of dextran sodium sulfate induced gut leakage and inflammation and stimulated δ-secretase in the guts of both wild-type and 3xTg mice, however, elevated Aβ and pTau in the colon as well as detection of Aβ in the cortex were only found in 3xTg mice in a C/EBPβ/δ-secretase-dependent manner [152]. Dextran sodium sulfate-induced cognitive dysfunction in 3xTg mice was alleviated either by knocking out C/EBPβ/δ-secretase or by performing vagotomy, both of which reduced AD pathologies and improved cognitive function [152]. Finally, the injection of Aβ, tau fibrils, or brain lysates from AD patients into the colon of mice, activated C/EBPβ/δ-secretase signaling, which transmitted signals from the gut to the brain via the vagus nerve, triggering Alzheimer's pathology and cognitive dysfunction [152]. Additionally, levels of bacterial amyloid curli were found to be elevated in Tg-AD mice, activating the vagus nerve and resulting in an increase in protein gene product 9.5 (PGP 9.5), a neuroendocrine marker. This underscores the significance of gut-vagus-brain signaling in response to bacterial amyloids [153].

These findings suggest a potential pathway through which gut-derived factors may influence neurodegenerative processes via vagus nerve activation, highlighting the importance of further research in understanding the intricate interplay between the microbiome, the vagus nerve, and brain health.

## Gut Microbially-derived Metabolites and Brain Function in ADRD

Host's diet provides many of the substrates that microbes use to thrive in the gut. Thus, microbiome composition and the metabolites it generates are directly impacted by diet. Dietary fiber contains carbohydrates that are not accessible to the host. Many gut dwelling microbes are capable of breaking down these substrates generating energy and biomass as well as producing metabolites such as SCFA. For example, bacterial taxa such as *Eubacterium*, *Faecalibacterium*, and *Roseburia* degrade fibers to produce butyrate, one of the main SCFA [154]. Patients with mild cognitive impairment (MCI) showed lower levels of gamma-aminobutyric acid (GABA)-producing microbes and GABA as well as higher levels of GABA-regulating microbes when consuming the modified Mediterranean ketogenic diet (Mediterranean diet with a low carbohydrate intake) compared with the American Heart Association diet (a diet that has an emphasis on low in saturated fat and cholesterol with limited sodium intake) [155]. Some observational and early clinical trial studies suggested that the Mediterranean-DASH Intervention for Neurodegenerative Delay (MIND) diet is associated with better cognition and reduced risk for cognitive decline [[156], [157], [158]], however, a multi-site clinical trial of the MIND diet did not find changes in cognition and brain MRI outcomes among cognitively unimpaired participants with a family history of dementia who followed the MIND diet compared to those who followed a control diet [159]. Although no microbiome results have yet been released in relation to the MIND diet, dietary intervention studies that are similar to the MIND diet such as the traditional Mediterranean diet have been shown to increase gut microbiome diversity [160,161] and SCFA production [162,163], and have been shown to lower the risk of depression [[164], [165], [166]] and improve cognitive function [89].

Taken together, these studies suggest that dietary components impact the composition of gut microbiome and the metabolites it produces which in turn can modulate host metabolism and have the potential to prevent, delay, and alleviate features of disease progression. In the following sections, evidence is reviewed suggesting that gut microbial metabolites are linked to ADRD. Precursor or dietary sources used by microbes to produce metabolites that impact host metabolism and physiology related to ADRD are described (Table 1).

**Table 1:** Summary of gut microbial metabolites associated with ADRD pathology.

Precursors or dietary components	Microbial metabolites	Potential impact on ADRD pathology^a^
Microbiota-accessible carbohydrates (e.g., dietary fibers)	Acetate	- Improved cognitive health in mice [167,168] - Reduced inflammatory signaling in astrocytes *in vitro* [169] - Salivary levels positively correlate with dementia [170] - Increased neuronal activation (c-FOS) in mice [171]
	Propionate	- Positive correlation with amnestic MCI [172] - Salivary levels positively correlate with dementia [170] - Dose dependent regulation of neuronal homeostasis [173] - Neuroprotective upon probiotic supplementation in 3xTg-AD mice [174] - Increased BBB tight junction expression *in* vitro [175]
	Butyrate	- Reduced astrocyte reactivity [176] - Lower amyloid SUVR uptake [177] - Decreased abundance of gut bacteria producing butyrate correlated with MCI [172] - Facilitated oligodendrocyte maturation in mice [178] - Reduced microglial inflammatory response [179] - The abundance of gut bacteria producing butyrate was negatively correlated with gray matter free water signal [180] - Increased BBB tight junction expression *in vitro* [175]
Primary BAs from cholesterol	Secondary BAs	- Negative association between conjugated secondary BAs:primary BAs ratios and CSF Aβ1–42 levels and neuroimaging markers, positive correlation between conjugated secondary BA and CSF p-tau and t-tau, and negative correlation between conjugated secondary BA and neuroimaging markers [28] - DCA:CA ratio positively correlated with cognitive decline [27] - Promoted higher intestinal permeability and inflammation [181]
Tryptophan derivatives from proteins	Kynurenine	- *Lactobacillus reuteri* produced neuroprotective kynurenic acid [182] - Kynurenic acid levels in CSF were higher in AD compared to controls and were associated with slower clinical progression of AD [183]
	Serotonin	- Diminished the effect of antidepressants on depressive-like behavior [184]
	Tryptamine	- Suppressed neuroinflammation in an AhR-dependent manner and promoted butyrate production in the murine model of multiple sclerosis treated with tryptamine [185]
	Indoles	- IPA prevented Aβ protein aggregation [[41], [42], [43]] - IPA attenuated neuroinflammation and enhanced neuronal survival [186] - IPA improved axonal regeneration and repair [187] - Elevated IS levels in the elderly with CKD and dementia [188] - IS induced neuropsychological symptoms and cognitive impairment [189]
Choline, betaine, l-carnitine	TMA/TMAO	- Positively correlated with aging in mice and humans [54,56,190,191] - Positively correlated with increased CSF phosphorylated tau and biomarkers of neurodegeneration in patients with AD and MCI [57] - Positively correlated with increased markers of astrocyte activation and proinflammatory cytokines in the brain in aged mice [56] - Caused impaired memory and learning, increased neuroinflammation, and increased markers of astrocyte activation in young mice [56] - Induced neuronal loss in AD mice [59]

a The contrasting outcomes for each metabolite are described in the following subsections.

### Short chain fatty acids (SCFA)

In this section, we focused primarily on acetate, propionate, and butyrate which are carboxylic acids that contain aliphatic tails of 2, 3, and 4 carbons respectively (Fig. 1 and Table 1). Excluding acetate, which is produced by both the host and the microbiome, butyrate and propionate are almost exclusively produced in the gut through bacterial fermentation of indigestible dietary fibers [[192], [193], [194]]. Multiple taxa from all phyla able to colonize the gut are capable of producing acetate. There are also several taxa capable of producing propionate including members of the Bacteroidota, Bacillota, and Verrucomicrobiota. In contrast, the production of butyrate is fairly restricted to taxa within the Bacillota, including species within the *Lachnospiraceae* and *Ruminococcaceae* families [24]. The amount of detectable SCFA in humans tapers within the gut from proximal to distal colon from 70-140 ​mmol to 20–70 ​mmol. Butyrate is a major energy source for colonic epithelium, this causes a significant reduction in its abundance beyond the intestine. Acetate can be synthesized by both the host and many gut bacteria. It is the most abundant SCFA in systemic circulation [195], but more research is needed to decouple the kinetics of host versus microbiome-produced acetate. SCFA are then transported via passive diffusion, bicarbonate exchange, Na^+^ coupled transport, or specific monocarboxylate transporters (MCT) [23] into host cells where they can impact host biology by inducing epigenetic modifications, and also initiate signaling cascades mostly by interacting with specific G-protein coupled receptors (GPCRs), also termed as free fatty acid receptors (FFARs) [23]. Circulating SCFA were also shown to be precursors of cholesterol and free fatty acids, and their excess is excreted through breath and urine [196].

SCFA were also shown to influence the host's metabolic, gastrointestinal, cardiovascular, and cerebrovascular health through various specialized, and non-specific interactions [197,198]. Although FFAR gene expression is observed throughout the system, the kinetics of SCFA-FFAR interactions specific to the brain remain veiled. It is also important to note that FFAR signaling pathways are not exclusive to SCFA; as GPR41, GPR43, and GPR109A have several other ligands that can trigger their signaling cascades [199]. SCFA can potentially have direct interactions with the CNS, there is evidence of relatively low concentrations of SCFA in CSF of human subjects (propionate at 2.8 ​± ​3.2 ​μM and butyrate at 0–2.8 ​μM) [200] as well as mice colonized with *Clostridium butyricum* [201] indicating that SCFA can, in fact, cross the BBB. However, more information is needed to identify supplementation methods required to reach the concentrations necessary to achieve therapeutic activity.

SCFA may be increased via gut microbiome alterations and/or by providing high levels of the substrates that support their production [202,203]. Furthermore, many studies have also shown that SCFA levels can be increased systemically via oral supplementation of sodium salt forms such as sodium acetate, sodium propionate, and sodium butyrate [[204], [205], [206], [207]]. Esterified versions of SCFA can also be supplemented effectively in a prodrug form, in which the SCFA is esterified to a glycerol backbone; e.g., tributyrin [208] and tripropionin [209]. These compounds may better recapitulate the pharmacokinetics of microbial SCFA production as they are absorbed more distally. However, their effects on AD need to be further explored.

Measurements of SCFA in a small cohort of patients with MCI/AD showed significant differences in their abundance between AD and healthy controls [172]. Individuals consuming a modified Mediterranean ketogenic diet and the American Heart Association diet exhibited significant differences in gut microbiome composition and SCFA production, alongside associated trends in AD biomarkers suggesting potential beneficial effects of SCFA. These associations point toward potential dietary intervention methods to induce alterations in the gut metabolome [210]. In a study involving 89 individuals ranging between cognitively unimpaired to dementia diagnoses, butyrate negatively correlated with amyloid standardized uptake volume ratio (SUVR) [177], this corroborated previously identified associations suggesting that butyrate promotes health. Investigating free water levels as a proxy of neuroinflammation in gray matter through diffusion-weighted neuroimaging showed strong associations with a decreased abundance of gut bacteria that produce butyrate in subjects with higher signals [180]. However, a study comparing individuals with dementia to age- and sex-matched controls showed significantly increased concentrations of acetate and propionate in the saliva of subjects with dementia [170]. Pre-onset amnestic MCI and dementia patients showed progressively decreasing levels of SCFA, and an increased abundance of *Bacteroides*, which mostly produced propionate and acetate [172]. It is important to note that these association studies do not use CSF levels of these. The above results suggest that butyrate might be potentially beneficial to AD patients whereas results for acetate and propionate are conflicting (Table 1). Furthermore, the available association data suggests an interesting contrast between measurements of SCFA in plasma, saliva, and feces; while fecal SCFA might be more indicative of their production, plasma and salivary SCFA levels provide insights about their bioavailability beyond the liver [211].

The neurovascular unit involves endothelial cells, astrocytes, pericytes, smooth muscle cells, neurons, and microglia; it aids in maintaining the hemodynamics, CSF flow, and metabolism within the brain parenchyma. Identifying specific roles of gut-microbial metabolites in modulating these individual elements *in vitro* would allow us to tease novel causal links in host-microbe interactions with cytological specificity. SCFA were shown to influence the brain vasculature as well as the three components of the ATN hypothesis. *In vitro* studies showed that butyrate and propionate modulate mitochondrial networks and actin cytoskeletal arrangement in brain endothelial cells, protecting them from LPS [175]. Astrocytes showed sexually dimorphic, dose-dependent responses to physiologically relevant concentrations of acetate (150 ​μM, 750 ​μM, and 1.5 ​mM), propionate (3.5 ​μM, 17.5 ​μM, and 35 ​μM), and butyrate (2.5 ​μM, 12.5 ​μM, and 25 ​μM). Interestingly, butyrate elicits a response only in astrocytes derived from female mice whereas both acetate and propionate induced transcriptional differences in astrocytes derived from male mice [169]. Another study shows SCFA provided in a 3:1:1 ratio (acetate, propionate, and butyrate) reduces the reactivity of primary astrocytes via SGK1/IL-6 (serum and glucocorticoid-regulated kinase 1/Interleukin 6) signaling post hypoxia, which enables them to be protective toward oligodendrocyte precursor cells [212]. Intragastric administration of acetate to APP/PS1 mice attenuated cognitive impairment and reduced CD11b, a microglial marker for inflammation, testing acetate on microglia *in vitro* resulted in upregulated GPR41 and downregulation of ERK/JNK/NF-κB (extracellular signal-regulated kinase/c-JUN N-terminal kinase/nuclear factor kappa B) signaling cascade [167]. Primary microglia from inulin-fed mice show attenuated production of TNF-α, indicating a lesser degree of inflammatory response. When sequenced, microglia showed no expression of major GPCR for SCFA; FFAR2 or FFAR3, but expressed MCT1 and MCT4 transporters; however, inhibition of the transporters did not interfere with the anti-inflammatory phenotype, indicating that SCFA might interact with intracellular signaling mechanisms in these cells by crossing the plasma membrane through passive diffusion [213]. N9 microglial cells generated an inflammatory response with both pre-treatment and simultaneous treatment of sodium butyrate under an LPS challenge model, whereas primary microglial cells seem to reproduce anti-inflammatory properties similar to what was observed in other studies [179]. This surprising difference in transcriptional landscapes between cell lines and primary cells suggests that the origin of microglia within a system links strongly to their response toward SCFA. Antibiotic-mediated suppression of gut microbiome in wild-type mice undergoing cuprizone treatment elicits a significantly reduced myelination compared to mice with unaltered microbiome, indicating a role of microbial factors in myelin content. Following up with a mixture of acetate, propionate, and butyrate as well as treatment with individual SCFA within the same model showed significant improvement in mice treated with the mixture and butyrate but not acetate and propionate individually. However, microglia did not seem to play a role in the observed phenotype in this study. Instead, in organotypic slice cultures at 200 ​μM, which is significantly higher than observed concentrations that have been reported in the brain, butyrate seemed to influence oligodendrocyte maturation through HDAC inhibition similar to the effects of trichostatin A [178]. Interestingly, studying microglial profiles after treatment with SCFA indicated that the beneficial effects of these metabolites might be a result of synergistic action of mixtures of different SCFA instead of a single SCFA eliciting the effects [214]. A significant reduction in senescence-related gene expression was observed in microglia isolated from mice fed with a high-fiber diet; as an effect of the diet, these mice also harbored significantly higher concentrations of SCFA in the cecum [215]. This study adds to the differences observed in inflammatory response by microglia obtained from mice fed with inulin. Since pre-treatment with sodium butyrate did not alter primary microglia within the context of inflammation as compared to simultaneous treatment, these studies pose an interesting question about acute versus chronic exposure to SCFA in their therapeutic potential [215]. Propionic acid elicits a dose-dependent alteration in firing frequency of primary cortical neurons, however, the higher doses tested within this study are outside of the physiologically relevant concentration in the brain [173]. These *in vitro* studies highlight interesting phenotypes exhibited by different cell types under isolated conditions that lack cellular heterogeneity, since neurons, glia, and brain vasculature participate in a continuous signaling crosstalk, more evidence in more complex systems and at physiological concentrations is needed.

Microbial depletion and recolonization with microbiome from specific pathogen-free mice through two oral gavages showed improved BBB integrity in AD mice. These effects were associated with SCFA levels [216], corroborating the results found *in vitro* on endothelial cells. Supplementation of APP^NL−G-F^ mice with a probiotic (VSL#3) increased the expression of the neuronal activity marker c-FOS alongside circulating levels of lactate and acetate [171]. Supplementation of fermentable oligosaccharides was shown to increase SCFA levels [217], and alleviated amyloid plaque burden, reactive oxygen species, and improvement of behavioral health in 5XFAD mice, indicating a potentially new therapeutic avenue. Another study showed that the positive effects of SCFA were mediated by GPR43, GPR41, and GPR109A receptors, where deletion of these receptors was associated with increased AD pathology through microglial dysfunction and loss of neurogenesis [218]. In contrast, oral supplementation of sodium acetate, sodium propionate, and sodium butyrate, to APP/PS1 germ-free mice increased amyloid-beta plaque load comparable to that of conventionally raised mice [219]. A high-fiber diet increased butyrate production and reduced propionate levels in transgenic AD mice that showed reduced glycolysis and mitochondrial respiration in reactive astrocytes. Treatment of mice with antibiotics inhibited the beneficial effects of high-fiber diet [176]. Exposure of 5XFAD mice to sodium butyrate via drinking water, reduced Aβ levels, and promoted synaptic plasticity as well as improved cognitive performance [220,221]. Another study showed almost no changes in AD phenotypes following exogenous supplementation of an SCFA mixture. Comparing results among different studies using similar supplementation methods, suggested that the time at which SCFA are dosed, the concentrations at which they are administered, and the gut microbial composition of the mice treated with these metabolites may play an important role in the effects they elicit in the host [222]. *Lactobacillus plantarum* PS128 supplementation in 3xTg-AD mice slows AD progression exacerbated by intracerebroventricular streptozotocin injection through propionate, GSK3β, and gliosis [174]. A study performed by co-housing AD mice with wild-type mice showed AD-associated gut dysbiosis in wild-type mice and these changes were reversed by oral gavage of *Lactobacillus* and *Bifidobacterium* through butyric acid-mediated acetylation of GSK3β and its subsequent activity as tau kinase. This study also showed that partners of AD patients developed similar AD-associated gut dysbiosis when living in the same household [223]. Exposure to exogenous SCFA increased glial reactivity and tau pathology in transgenic apolipoprotein E (ApoE)-tau mice, this phenotype is not observed in germ-free mice or mice with antibiotic-ablated gut microbiome, hinting causal negative associations with respect to tau pathology through neuroinflammation-neurodegeneration axis [224]. These studies provide fascinating insights into the delivery methods of SCFA, the site of bioavailability to host, and unmapped interconnected host-microbe systems that dictate pathophysiological roles of SCFA specific to the experimental models.

In summary, evidence to date suggests that SCFA can have multiple albeit differing effects on AD-related phenotypes. This conflicting evidence may be due to possible confounding factors including pharmacokinetics (produced in the gut-produced vs oral delivery), dose, delivery methods, techniques to induce or upregulate production, gut microbial community composition, experimental AD model, and biomarkers measured. Highly controlled gnotobiotic studies using defined synthetic communities of known metabolic activity could potentially help to address some of these limitations.

#### Less abundant short chain fatty acids in ADRD

Although acetate, propionate, and butyrate are the most abundant SCFA, there may be roles for less abundant SCFA in ADRD. One study found that in addition to these three SCFA, fecal isobutyrate, isovalerate, valerate, and hexanoate were decreased in patients with AD [172]. Other studies found that isobutyrate was inversely associated with cognitive decline [225], and that serum isobutyrate levels are increased in an APP^NL−G-F^ AD mouse model [171]. A report in humans showed that AD patients have increased fecal hexanoate [226]. Studies in rats have also found beneficial effects of valerate on oxidative stress, neuroinflammation, and autophagy pathways in a PD model [227], as well as in dementia [228]. One study found that valerate-treated rats had reduced plasma Aβ-42 [229]. However, findings regarding these less abundant SCFAs are conflicting; another study found that fecal isovalerate and isobutyrate levels were increased in participants with dementia [230]. Further investigation of these less abundant SCFA may provide further insights into their potential role in ADRD.

### Bile acids (BAs)

Bile acids (BAs) are host-derived and microbial-modified amphipathic steroid molecules produced from cholesterol in the liver. Humans produce two bile acids, cholic acid (CA) and chenodeoxycholic acid (CDCA)—i.e., primary bile acids, which are conjugated with glycine (prevalent in humans) or taurine (prevalent in rodents) in the liver and stored in the gallbladder. Conjugated BAs (a.k.a. bile salts) are the main components of the bile which is released into the duodenum upon consumption of a meal to emulsify fats for lipid digestion and absorption [231]. Once in the intestine, BAs can be metabolized by gut microbes through different reactions including deconjugation, dehydroxylation, epimerization, and dehydrogenation, to produce unconjugated and secondary BAs. Prominent secondary BAs in humans include deoxycholic acid (DCA) and lithocholic acid (LCA), which are mainly generated via bacterial 7α-dehydroxylation [26] (Fig. 1). Conjugated and deconjugated BAs are reabsorbed (∼95 ​%) in the distal ileum and recycled by the enterohepatic circulation or excreted (∼5 ​%) in the feces where they are intensively metabolized. The secondary BAs that are reabsorbed can be re-conjugated with glycine or taurine such as taurodeoxycholic acid (TDCA) and taurolithocholic acid (TLCA).

Altered BA profiles have been linked to AD pathology. The increases in microbially derived secondary BAs were significantly associated with amyloid, tau, and neurodegeneration (A/T/N) biomarkers for AD [28]. An increase in the DCA:CA ratio has been strongly correlated with cognitive decline [27]. Another study showed elevated blood concentrations of ammonia and conjugated BAs in cirrhotic patients with hepatic encephalopathy (HE), a neuropsychiatric abnormality observed in patients with chronic liver disease [232]. This observation was confirmed in a murine model, which showed increased levels of ammonia and BAs in the blood and brain induced by the elevation of apical sodium-dependent BA transporter (ASBT)-mediated BA reabsorption [232]. Multiple lines of evidence showed that the gut microbiota can modulate BA profiles. *Ruminococcus gnavus* was identified to potentially contribute to ursodeoxycholic acid (UDCA) formation in the human colon [233]. The inoculation of *Bilophila wadsworthia* in mice fed with a high-fat diet synergized to alter the functional potential of gut microbes and bile acid metabolism and promote higher intestinal permeability and inflammation [181]. These effects were inhibited by the probiotic strain *Lactobacillus rhamnosus* CNCM I-3690 reverting the host dysfunction. Particularly, a higher abundance of the genera *Dorea* and *Sutterella* and species *Ruminococcus gnavus* was observed in high-fat diet-fed mice [181].

A small number of studies have examined the relationship between gut microbiome, bile acids, and psychological disorders or ADRD (Table 1). The abundance of *Ruminococcus gnavus* was higher in Crohn's disease patients with psychological disorders. The relative abundance of *Ruminococcus gnavus* was positively associated with the Self-Rated Anxiety Scale (SAS) and Self-Rated Depression Scale (SDS) [29]. Fecal levels of bile acids including TDCA and TLCA were negatively correlated with *Ruminococcus gnavus* and positively correlated with SAS [29]. A study on a transgenic rat model of AD exposed to traffic-related air pollution (TRAP) observed increases in multiple characteristics related to AD pathology including amyloid plaque deposition and hyperphosphorylated tau levels as well as neuronal cell loss and cognitive deficits [234]. A follow-up study using the same rat model exposed to TRAP showed modulation of BA concentration and increased *Turicibacter* and *Ruminococcus gnavus* in wild-type and transgenic female rats [235].

In summary, there is a reciprocal crosstalk between gut microbes and BA profile that impacts myriad aspects of host physiology and disease. Emerging evidence is linking BA specific profiles with AD-associated phenotypes. Larger human studies are needed to validate these initial findings. Additional mechanistic studies are also needed to establish how BA impact disease.

### Tryptophan derivatives

Tryptophan is one of the essential amino acids that cannot be synthesized in the body requiring its supply from the diet. Tryptophan is essential for protein biosynthesis and thus important for the growth and maintenance of proteins in the body as well as for the synthesis of other biological compounds including enzymes, coenzymes, and neurotransmitters. The free tryptophan in the blood is the main contributor to protein synthesis in the host [236]. Tryptophan is a precursor for serotonin (5-hydroxytryptamine; 5-HT) and melatonin synthesis. Serotonin is a neurotransmitter mainly produced in the enterochromaffin cells in the gut regulating mood, sleep, digestion, and wound healing [237]. Melatonin is a hormone produced by the pineal gland in the center of the brain controlling the circadian rhythm and sleep-wake cycle [238]. The synthesis of serotonin in the CNS is needed to produce melatonin. The other fate of tryptophan is its oxidation via the kynurenine pathway (∼90 ​%). This pathway occurs in multiple organs such as the liver, gut, and brain as well as immune cells converting tryptophan to NAD^+^ for energy demands [239] or to other intermediate kynurenines such as kynurenic acid and quinolinic acid [240].

As described, the conversion of tryptophan to its metabolites can occur in the gut where gut microbes reside and it is suggested that gut microbiota are involved in the regulation or synthesis of tryptophan derivatives generating metabolites including kynurenine, serotonin, tryptamine, and indoles (Fig. 1). The potential role of these metabolites in the context of the gut-brain axis related to the pathophysiology of ADRD is further discussed (Table 1).

#### Kynurenine (kynurenic acid, quinolinic acid)

Tryptophan is metabolized to kynurenine via the rate-limiting enzyme indoleamine-2,3-dioxygenase 1 (IDO1) or tryptophan 2,3-dioxygenase (TDO). While IDO1 is expressed in various organs (liver, gut, and brain), TDO is expressed solely in hepatocytes [241]. Aging has been associated with enhanced neuropsychiatric disorders due to the impairment of serotonin and melatonin synthesis [31,242,243]. Central tryptophan metabolism was reported to increase with age in the CSF of women and elevation of neurotoxic quinolinic acid was observed in these women [244]. Additionally, colonic epithelial cells from mice colonized with *Clostridium* species expressed a high level of IDO1 [245]. Investigators have suggested that Clostridia may activate colonic epithelial cells to produce molecules that induce T_reg_ cells and TGF-β [245]. Another study showed that the gut microbiome is involved in the IFN-γ-regulated expression of the *IDO* gene [246]. Moreover, IFN-γ limited the growth of *Salmonella* by enhancing the antimicrobial activities of intestinal epithelial cells and macrophages [246]. The gut microbiome shifted in pregnant mice accompanied by IDO1-dependent kynurenine production, intestinal inflammation, and pregnancy-associated insulin resistance, which were reversed in IDO1-knockout [247]. Therefore, accumulating studies suggest that gut microbiome can modulate IDO1 expression level which may lead to alterations of diverse kynurenines and host phenotypes including immunomodulation and inflammation related to disease severity known to affect brain health (Fig. 1). Increased levels of quinolinic acid exert excitotoxic effects, whereas higher levels of kynurenic acid exhibit neuroprotective effects which are influenced by the gut microbiota [32,33]. Defects in the kynurenine pathway that lead to more neurotoxic effects have been reported to be associated with depression, schizophrenia, Huntington's disease, and AD [32,33,240,248,249]. One study showed higher kynurenic acid concentrations in CSF of AD patients compared to cognitively unimpaired controls and higher kynurenic acid levels were associated with slower progression of AD [183]. However, the evidence is scarce in humans connecting microbiome, kynurenine, and ADRD pathology. Further clinical trials and observational studies are needed to investigate the gut microbiome relationship with ADRD in the kynurenine pathway and metabolites.

#### Serotonin

Serotonin (5-HT) is synthesized from tryptophan which is dependent on a rate-limiting enzyme tryptophan hydroxylase (TPH) (Fig. 1). Serotonin, a neurotransmitter, is produced in small proportions in the CNS and larger amounts (∼90 ​%) in the enterochromaffin cells of the gut [250]. Peripheral serotonin does not cross the BBB under normal physiological conditions. Two isoforms of TPH, i.e. TPH1 and TPH2, are primarily expressed in enterochromaffin cells and neurons, respectively [251]. Evidence supports that disrupted global serotonergic neurotransmission is consistently observed in dementia and associated with behavioral and psychological symptoms of dementia [252,253]. Lower levels of serotonin in parts of the brain of adults with MCI were reported [254]. Another study showed reduced serotonin transporter availability in MCI male adults and lower cortical serotonin transporters were associated with worse auditory-verbal and visual-spatial memory test scores in these adults [255]. Studies have demonstrated serotonin deficits or serotonergic changes in multi-infarct type of vascular dementia [256], Pick's disease (a.k.a. frontotemporal dementia) [257], and Lewy body dementia [258,259]. In animal models, chronic anxiety states in rats were associated with dysregulation of TPH2 expression in the brain, suggesting the potential link between psychological disorder and CNS serotonin production [260]. It is crucial to note that multiple serotonin receptor subtypes (e.g., 5-HT_1A_, 5-HT_1B_, 5-HT_2A_, and 5-HT_3A_) might have different roles, and alterations in these receptors were suggested to impact the maturation of selected brain circuits [261]. For instance, the effects of antidepressants on neurogenesis and behavior were blocked after knockout on the 5-HT_1A_ receptor in mice brains including the hippocampus [262]. One of the most widely expressed 5-HT receptors in the brain is 5-HT_2A_. It is known to be involved in late neuronal maturation and differentiation [263,264].

Gut microbiome is known to impact serotonin production in the gut (Fig. 1). Studies have suggested that gut microbiota may induce transcription of the *TPH1* gene where they found decreased expression of *TPH1* in colons of germ-free mice [35] as well as increased colonic mRNAs TPH1 in humanized and conventionally raised mice [34]. In mice treated with antidepressants reduced abundance of genera *Ruminococcus*, *Adlercreutzia*, and unclassified *Alphaproteobacteria* [184]. Particularly, the effects of an antidepressant (duloxetine) were diminished after being treated with *Ruminococcus flavefaciens* [184]. Down-regulation of genes involved in neuronal plasticity was observed in cortical gene expression induced by *Ruminococcus flavefaciens* treatment [184]. Treatment with escitalopram in major depressive disorder patients showed a tendency to restore composition of gut microbiota similar to healthy controls [265]. Interestingly, *Ruminococcus* was also positively correlated with plasma levels of IL-1β, lipid metabolites, and clinical parameters in major depressive disorder patients whose gray matter volumes were significantly decreased compared to healthy controls [37]. As abnormalities in serotonin levels are associated with depression and depression is a risk factor for developing ADRD [36,38], it is crucial to find mechanisms that gut microbes contribute to serotonin production and its related genes that could influence the host's brain physiology and neurologic functions.

#### Tryptamine

Tryptamine is produced from tryptophan by gut microbes via tryptophan decarboxylase (TrpD) activity. An *in vitro* study using *Clostridium sporogenes* and *Ruminococcus gnavus* showed their ability to decarboxylate tryptophan to tryptamine [266]. This study also confirmed the presence of tryptophan decarboxylase in at least 10 ​% of the samples from the Human Microbiome Project [266]. The same group has identified that tryptamine can modulate colonic secretion mediated by the epithelial 5-HT_4_ receptor in mice [267]. Tryptamine, an AhR ligand, administration in the murine model of multiple sclerosis suppressed neuroinflammation in an AhR-dependent manner and promoted butyrate production, suggesting that gut microbiota (*Dehalobacterium*, *Bacteroides*, and *Peptostreptococcaceae*) plays an essential role in maintaining host's intestinal homeostasis [185]. Conversely, gut microbially derived tryptamine may induce inhibition of tryptophanyl-tRNA synthetase which is associated with vasculopathies in AD brain [268,269]. Tryptamine is shown to cross the BBB [80], however, it is still uncertain whether gut microbially derived tryptamine impacts neurological functions and ADRD.

#### Indoles

Gut microbes are capable of directly transforming tryptophan into indoles [270] (Fig. 1). Multiple indole derivatives including indole-3-acrylic acid (IA), indole-3-acetic acid (IAA), indole-3-aldehyde (IAld), indole propionic acid (IPA), indole-3-lactic acid (ILA), and indoxyl-3-sulfate (IS) are also produced from tryptophan by gut microbes [270,271]. For example, indole is generated from tryptophan by the tryptophanase (TnaA), an enzyme expressed in multiple gram-positive (*Clostridium* spp.) and gram-negative (*Escherichia coli* and *Bacteroides* spp.) bacteria. Multiple enzymatic reactions including aromatic amino acid aminotransferase (ArAT), ILA dehydrogenase and dehydratase, and acyl-CoA dehydrogenase are performed by gut microbes such as *Clostridium sporogenes* [272,273] as well as *Clostridium cadaveris* and *Peptostreptococcus anaerobius* to produce IPA [273]. The importance of these indole derivatives lies in their interaction with the host's metabolism and signaling. ILA has been identified to have anti-fungal and anti-bacterial activities [274,275]. Many indole derivatives act as ligands for AhR, a transcription factor that is associated with immune regulation and is expressed in immune cells [276]. Additionally, IAld-producing lactobacilli (*Lactobacillus reuteri*) catabolizes tryptophan and produces IAld which contributes to AhR-dependent regulation of IL-22 [277]. IL-22 provided resistance to fungal colonization as well as mucosal protection from inflammation [277]. IPA was found to maintain mucosal homeostasis via the pregnane X receptor (PXR) by increasing the expression of junctional protein-coding mRNAs while decreasing TNF-α [39,40]. Moreover, mice fed a high-fat diet showed reduced intestinal permeability after IPA supplementation [278]. Given that gut barrier function and gut immunity may influence the gut-brain axis in ADRD, the role of indoles need to be further examined in the context of ADRD.

IPA is one of the indoles suggested to be neuroprotective. IPA showed anti-aggregation activity preventing Aβ protein aggregation [[41], [42], [43]]. Indolepropionamide (IPAM), a synthesized indole substance from adding an amide group to IPA, was reported to reduce reactive oxygen species (ROS), modulate age-dependent decline of mitochondrial metabolism, and increase the lifespan of rotifer [279]. As indoles are known to cross the BBB, administration of tryptophan indeed increased levels of IPA in the brain [279]. Higher levels of circulating IPA were positively associated with alpha-diversity measures of gut microbiome even after controlling for dietary fiber intake [280]. IPA was also found to modulate the secretion of glucagon-like peptide-1 (GLP-1) from enteroendocrine L cells as well as to exert anti-oxidative stress capacity, suggesting a potent protective effect against type 2 diabetes, one of the risk factors for developing dementia [281]. The serum levels of IPA were lowered in a mouse middle cerebral artery occlusion (MCAO) model [186]. IPA treatment in male mice with MCAO reshaped the microbial community similar to the control group [186]. Administration of IPA not only improved intestinal barrier function but also attenuated neuroinflammation and enhanced neuronal survival by regulating immune functions [186].

Indoxyl sulfate (IS), on the other hand, is an indole derivative that has renal toxicity in high concentrations [282,283]. Indole is absorbed and metabolized in hepatocytes into IS which were elevated in patients with chronic kidney disease (CKD). IS has been associated with cardiovascular disease in patients with CKD [284]. Parameters of renal dysfunction were positively correlated with levels of IS as well as with the prevalence of cardiovascular events in patients with CKD [285]. IS relationship with complications of cardiovascular and kidney diseases extends to dementia. IS may cross the BBB and activate astrocytic AhRs [286]. A study that measured serum IS levels in non-demented and demented elderly with CKD showed elevated IS levels in the elderly with dementia, suggesting that monitoring the serum levels of IS may be important to prevent the possible influence of IS and its accumulation in the brain that could exacerbate progression of dementia [188]. Intraperitoneal administration of IS in mice subjected to unilateral nephrectomy showed accumulation of IS in the blood, prefrontal cortical tissues, and CSF compared to nephrectomized mice administered with normal saline. IS-administered mice exerted neuropsychological symptoms as well as cognitive impairment proposing pathological roles of IS in the CNS [189]. Consistently, patients with CKD showed higher levels of IS and lower cognitive function, which were associated with poorer executive functions only in early-stage CKD patients [287]. An *in vitro* study investigated the effect of IS on CNS cells and showed that IS increased AhR and induced activation of NF-κB, ROS, and pro-inflammatory cytokine production, while downregulated cell-protective factors in glial cells [288]. Additionally, necrotic neurons and hyperplastic glial cells as well as markers of oxidative stress and inflammation in the brain histology were observed in mice treated with IS [288].

As suggested by multiple preclinical and clinical studies, gut microbially-derived indole and its derivatives have potential implications for brain function and disease that may be modulated by the composition and function of microbes that are uniquely responsible for producing diverse indoles via tryptophan catabolism (Table 1). Further research with manipulation of gut microbiome composition and function is needed to understand their impact on the production and utilization of diverse indoles and the pathophysiological action of indoles.

### Trimethylamine/trimethylamine N-oxide (TMA/TMAO)

Trimethylamine N-oxide (TMAO), a metabolite whose production depends on gut microbial activity, has deleterious effects on disease [45,49]. Gut microbial metabolism of quaternary amines including choline, betaine, and carnitine produces trimethylamine (TMA) [44,45] (Fig. 1). TMA is absorbed into the portal circulation and transported to the liver, where flavin monooxygenases, particularly flavin monooxygenase 3 (FMO3), metabolize TMA to produce TMAO [44,45] (Fig. 1).

Choline is found in foods such as fish, beef, eggs, cheese, nuts, beans, and poultry [49,289,290]; it is an essential nutrient, necessary for biological activities such as the synthesis of membrane phospholipids and neurotransmitters [291]. Choline can be synthesized endogenously, but this does not meet the levels necessary for optimal health, particularly during pregnancy [49]. Although TMA is primarily produced from choline, it can also be produced from l-carnitine, found in red meats [46], and betaine, found in beets, spinach, whole grains, and seafood [292].

There are three enzyme complexes that gut microbes can use to produce TMA. Choline TMA-lyase (cut C/D) is primarily used [293]. Several gut microbes are capable of producing TMA from choline, primarily from the Bacillota (Firmicutes) and Pseudomonadota (Proteobacteria) phyla, including *Desulfovibrio desulfuricans*, *Anaerococcus hydrogenalis*, *Clostridium asparagiforme*, *Clostridium hathewayi*, *Clostridium sporogenes*, *Escherichia fergusonii*, *Proteus penneri*, *Edwardsiella tarda*, *Providencia rettgeri*, and *Providencia rustigianii* [49,293]. Most of these strains have a microbial gene cluster that encodes genes for conversion of choline to TMA; these include *cutC*, which encodes a glycyl radical enzyme with choline TMA-lyase activity; *cutD*, which encodes a glycyl radical-activating protein; and genes encoding proteins involved in the assembly of microcompartments, which may sequester the acetaldehyde byproduct [45,49]. Choline undergoes a radical carbon-nitrogen bond cleavage by TMA-lyase, leading to the production of TMA [45,49]. To convert carnitine to TMA, bacteria species including *Acinetobacter calcoaceticus* and *Serratia marcescens* use the two-component Rieske-type oxygenase/reductase (cntA/B) [293]. To convert betaine to TMA, gut bacteria use another two-component Rieske-type oxygenase/reductase (yeaW/Y) [293]. After TMA is transported by portal circulation to the liver, FMO enzymes in hepatocytes catalyze the oxidation of TMA to TMAO [44,49] (Fig. 1). While both FMO1 and FMO3 produce TMAO [44,290], FMO3 is significantly more active in the production of TMAO [44].

In humans, upon entering circulation TMAO reaches micromolar concentrations in blood [59,294]. Most TMAO is excreted within 24 ​h by the kidneys [295]. Healthy humans have plasma TMAO levels of about 0.5–5.0 ​μM [190,296,297], while these levels can increase to about 40 ​μM in renal failure patients [296,297]. In mice, circulating TMAO levels are significantly higher in females [[296], [298]]. This sex difference is not observed in humans [51,298]; this may be due to dietary factors obscuring differences. Testosterone or androgens appear to repress FMO3 expression and TMAO production, as shown by the comparison of mice with intact testes and gonadectomized mice [298,299]. Parallel studies found a correlation between estrogen and FMO3 expression [298,299]. TMAO has effects on a variety of tissues, many of which exacerbate disease [49,300].

Although studies have found a positive correlation between consumption of TMAO precursors and cognition [[301], [302], [303]], increased TMAO levels have been found to be associated with a variety of diseases [51,57,296] affecting well-vascularized organs such as the heart, liver, kidneys, and brain. These include heart failure [304], chronic kidney disease [305], liver disease [306,307], tumors [308], obesity [309], diabetes [310], atherosclerosis [51,296], and AD [57]. TMAO has effects on a variety of tissues, leading to largely harmful effects including modulation of cholesterol and bile acid metabolism, promotion of platelet hyperreactivity [50], impairment of insulin signaling, and induction of endothelial and neuronal senescence [54,55]. TMAO promotes forward cholesterol transport, inhibits reverse cholesterol transport [296], and increases lipogenesis and bile acid synthesis [311]. TMAO has been found to impair glucose tolerance and insulin signaling and promote adipose tissue inflammation in mice on a high-fat high-sugar diet [312]. TMAO also activates the NLRP3 inflammasome [52,53] and contributes to vascular inflammation [52,53]. This can induce vascular calcification [52,53], or mineral buildup in arteries and veins, and lead to endothelial dysfunction. TMAO has been found to increase oxidative stress, accelerating endothelial cell senescence and vascular aging [54]. In mouse models of atherosclerosis, it was found that l-carnitine supplementation increased TMAO levels and atherosclerotic lesion size [313].

TMAO has also been found to have deleterious effects on the brain. TMAO is able to cross the BBB [57,294]. In the brain, TMAO induces neuronal senescence, increases oxidative stress, impairs mitochondrial function, and inhibits mammalian target of rapamycin (mTOR) signaling [55]. Plasma TMAO levels increase with aging in both mice [54,56,191] and humans [56,190]. TMAO levels also increase in whole brain lysates with aging in mice [56]. This contributes to brain aging and cognitive impairment. It has been found that increased TMAO levels in aged mice lead to increased levels of proinflammatory cytokines in the brain and markers of astrocyte activation [56]. It was found that TMAO given to younger mice caused impaired memory and learning, increased proinflammatory cytokine levels in the brain, and increased markers of astrocyte activation [56]. In human astrocyte cells, it was found that TMAO has direct effects on astrocyte activation [56]. It is possible that TMAO may influence neurodegenerative disease via its effects on glia and neuroinflammation (Fig. 1). It was also found that TMAO enhanced oligodendrocyte pyroptosis in the brains of rats, leading to demyelination [314]. Additionally, TMAO may cause BBB disruption by reducing the expression of tight junction proteins [315,316]. However, one study found that TMAO has a protective effect on BBB integrity. Further investigation is needed to disentangle the impact of TMAO on the BBB [317].

TMAO effects on AD specifically have also been explored. In a study employing a network-based ranking algorithm, it was found that among microbial metabolites known to be associated with AD in humans, TMAO ranked as the top metabolite that has genetic commonality with AD [318]. Human cohort studies found that CSF TMAO levels are increased in AD and MCI patients, and correlated with increased levels of CSF pTau and biomarkers of neuronal degeneration [57]. In contrast, plasma levels of TMAO were not associated with cognition, neuroimaging markers, or incident dementia, whereas levels of plasma choline were associated with lower cognition, lower total brain volume, and a higher white matter hyperintensity volume [319]. In a 5XFAD amyloid AD mouse model, TMAO supplementation in drinking water drives neuronal loss [59]. In APP/PS1 AD mice, it was also found that TMAO is associated with and may lead to increased levels of apolipoprotein J (or clusterin, CLU), a risk factor for AD, which increases the activity of β-secretase, which is involved in amyloid accumulation, promoting cognitive impairment [58,60].

## Gut Microbial Metabolite Modulation as Therapeutic Targets for ADRD

The gut microbiome has the potential to modulate diverse mechanisms in the host that are associated with healthy aging [64], risk reduction of developing neurodegenerative disorders [320], and prevention or slowing of further deterioration of cognitive [321] and behavioral [322] symptoms. Multiple lines of evidence show that gut microbiome modulation can be targeted as therapies for ADRD as well as other diseases, which aim to (1) recover gut microbiota composition similar to that of healthy individuals, (2) increase diversity and composition of beneficial microbes and their metabolites, (3) lower levels of detrimental microbes and their metabolites, (4) reinforce gut barrier integrity and function as well as gut immunity, and (5) minimize the risk of onset and/or progression of disease that could be triggered by the alteration of gut microbes.

Research on the gut microbiome in AD has shown that gut microbiota composition is altered in people with AD dementia and that the abundance of certain bacteria is associated with AD pathology, even among people who have not yet developed dementia [16]. Increased calprotectin, a marker for intestinal inflammation, is also associated with age and AD pathology (clinical status of AD, amyloid burden as shown with amyloid PET, and CSF biomarkers of AD and neurodegeneration) [323]. This suggests that gut microbiome composition and immunity may be associated with AD pathology, possibly even prior to over dementia symptoms. In addition, dysfunction of the gut-brain axis has been implicated in multiple psychiatric disorders and behavioral changes, where the microbiome plays an important role in the communication between the gut and the brain [15,[324], [325], [326]]. To harness the benefits of gut microbiome modulation in ADRD, preclinical and clinical interventions that target the gut microbiome including diets, prebiotics, probiotics, synbiotics, postbiotics, and FMT have been tested as potential therapies for ADRD. Extensive reviews have documented the effectiveness of these therapies in brain function and AD [[320], [321], [322],[327], [328], [329]]. In the next section, the potential therapeutic roles of gut microbes and microbially-derived metabolites on ADRD are considered (Table 2).

**Table 2:** Summary of human studies on gut microbiome modulation in ADRD and its clinical outcomes.

Approach	Intervention or treatment	Clinical outcomes	References
Dietary interventions	Modified Mediterranean-ketogenic diet (MMKD)	- Fecal butyrate was increased overall after MMKD - Fecal butyrate was slightly increased in CN and MCI after MMKD - CSF pTau_181_ was positively correlated with genus *Sutterella* and the Shannon index in MCI and with family *Ruminococcaceae* in CN after MMKD - CSF Aβ42 was negatively correlated with phylum Tenericutes and family *Enterobacteriaceae* and positively correlated with family *Rikenellaeae* and genus *Parabacteroides* in MCI after MMKD	[330]
		- Reduced the inflammatory marker GlycA in MCI after MMKD - Altered the BCAA-associated gut microbiome in MCI after MMKD - Increased serum valine levels in MCI after MMKD	[210]
		- Lowered *Alistipes* sp. (GABA-producing microbes) and enriched *Akkermansia muciniphila* (GABA-regulating microbes) in MCI on the MMKD - Altered bile acid pool in MCI with curcumin in their diet	[155]
Probiotics/Prebiotics	*Lactobacillus rhamnosus* GG (LGG) ​+ ​inulin from chicory root extract	- More abundant *Lactobacillus* in the group fed the probiotic compared to the placebo group - Lower relative abundances of *Dehalobacterium* and *Prevotella* in MCI after probiotic administration showed positive trend with an improved cognitive score	[331]
	*Bifidobacterium bifidum* BGN4 and *Bifidobacterium longum* BORI	- The relative abundances of *Eubacterium*, *Allisonella*, Clostridiales, and *Prevotellaceae* decreased after probiotic - *Eubacterium* and Clostridiales showed negative correlation with serum BDNF level - Increased serum BDNF level after probiotic - Improvement in mental flexibility test and stress score after probiotic	[332]
	*Bifidobacterium longum* subsp. *infantis* BLI-02, *B. breve* Bv-889, *B. animalis* subsp. *lactis* CP-9, *B. bifidum* VDD08, and *Lactobacillus plantarum* PL-02	- Increased abundance of *Bifidobacterium*, *Lactobacillus*, *Ruminococcus*, *Clostridium*, and *Akkermansia* and decreased abundance of *Megamonas* in the probiotic group - Increased serum BDNF after probiotic - Trend in less cognitive deterioration in AD patients after probiotic	[333]
FMT	From a healthy 27-year-old man	- Performing FMT to alleviate *C. difficile* infection significantly increased SCFA and improved cognitive function in a 90-year-old dementia patient - Increased Shannon diversity and enriched gut microbes (e.g., Bacteroidales, Bacteroidia, *Tannerellaceae*, and Actinobacteria) after the FMT	[115]

### SCFA

It has been noted that SCFA are produced mainly from dietary fiber. However, the complexity of this process increases when *Lactobacillus* and *Bifidobacterium* genera use dietary fiber to produce substrates such as lactate and acetate, which can then be used to produce SCFAs such as butyrate by different bacteria, through a process known as cross-feeding. Hence, treatment with bacteria that enable SCFA production gains viability if the host diet is rich in fiber and has the right bacteria that produce SCFA in the gut.

A therapeutic avenue that impacts SCFA is via probiotic and prebiotic supplementations. SLAB51, a probiotic formulation of lactic acid bacteria and bifidobacteria, restructured gut microbiome composition, increased fecal SCFAs, influenced plasma concentration of inflammatory cytokines and key metabolic hormones, slowed cognitive decline, and reduced accumulation of amyloid beta aggregates in 3xTg-AD mice [334]. Interestingly, probiotics and prebiotic supplementation of fibers such as oligosaccharides, inulin, and resistant starch is not the only potential method to increase SCFA, for example, d-β-hydroxybutyrate supplementation can increase SCFA production; specifically, butyrate and increased relative abundance of *Coprococcus* [335]. In contrast, colonization of germ-free mice with *Roseburia intestinalis* resulted in increased serum levels of β-hydroxybutyrate [336]. Together, these results point toward a complex physiological and metabolic landscape of butyrate *in vivo*. Since β-hydroxybutyrate was shown to be beneficial in alleviating AD pathology in 5XFAD mice [337], the scope of using butyrate – β-hydroxybutyrate in combating AD presents an interesting novel avenue of microbially modulated ketogenesis. A clinical trial (ID: NCT05943925) is already recruiting patients with AD to test a probiotic intervention comprising *Lactobacillus acidophilus*, *Lactobacillus casei*, *Bifidobacterium bifidum*, and *Lactobacillus fermentum* (2 ​× ​10^9^ colony forming units (CFU)/g of each), whose results would elucidate utilization of daily probiotic supplementation in combating ADRD. Since butyrate production can be upregulated with specific bacteria, a similar study could be performed with gut bacteria that produce butyrate and bacteria that enhance butyrate production to identify if previous results shown *in vitro* and animal models can be reproduced in human subjects. Although human studies highlight the beneficial effects of butyrate in AD through associations, modes of delivery and supplementation require more work to identify toxicological interactions of butyrate with the host. A study involving oral supplementation of tributyrin successfully increased circulating levels of butyrate up to 0.5 ​mM [338], which is similar to the dosage required to show effects *in vitro.* Although there are methods to increase circulating concentrations of SCFA, the amount required to increase intracellular concentrations at target tissues is unknown [339] or whether raising SCFA to such levels has other unintended effects on the host. With data pointing toward synergistic effects of SCFA largely suggesting its beneficial activity, SCFA-based therapeutics demand more evidence to outweigh conflicting results observed in comparable studies.

### BAs

Various gut microbes were suggested to be involved in BA metabolism, deconjugation, oxidation, epimerization, dehydroxylation, esterification, and desulfation [340,341]. Among these microbes, probiotic bacteria *Lactobacillus* and *Bifidobacterium* have been identified to participate in BA deconjugation, a critical early step in BA metabolism. Particularly, food-associated *Lactobacillus plantarum* strains showed selective ability to alter BA metabolism [342]. Other studies have shown the impact of *Lactobacillus plantarum* strains on increased hepatic synthesis of BA and its excretion [343,344]. *Bifidobacterium longum* strains were identified to produce deconjugated bile salts [345]. *Bifidobacterium animalis* subsp. *lactis* was shown to adapt to BA and low pH environments by increasing the intracellular ATP and by inducing the membrane-bound F1–F0 ATPase activity [346]. It is suggested that *Lactobacillus* and *Bifidobacterium* strains containing BSH may also store primary and secondary BAs in their cytoplasm in addition to deconjugating primary BAs [[347], [348], [349]]. Reduced conversion of primary to secondary BAs in the colon may reduce colonic inflammation and possibly colorectal cancer risk. In AD patients, *Bifidobacterium* and *Clostridium* which have deconjugation activities via BSHs [350] were less abundant [16,351], while increased secondary BAs were associated with amyloid and tau AD markers [28]. Additionally, higher secondary BAs as well as glycochenodeoxycholate (GCDCA):CA ratio were identified in a post-mortem brain metabolomics analysis of AD patients compared to controls, suggesting that these BAs may be related to cognitive decline in AD [352]. As some BAs can cross the BBB and have been shown to impact the BBB permeability [353,354], it is reasonable to hypothesize that BAs impact neurologic functions related to activation of receptors and ion channels in the brain which may contribute to the development of ADRD [353]. In an AD mouse model, mice treated with a probiotic (VSL#3) for 8 weeks showed decreased intestinal permeability and inflammation as well as serum secondary BAs (DCA and LCA) with no effects on memory function or other markers for AD or neurodegeneration [355]. Conversely, there is evidence showing that BAs can have protective effects by preventing Aβ accumulation in AD [353]. While the role of bile acids (BAs) in ADRD is controversial in various studies, it is important to note that the metabolism of BAs varies among species making it difficult to interpret between animal and human studies [356]. Additionally, the production of BAs can be influenced by many other factors that vary between individuals, such as diet, gut microbiome, and metabolic functions. Whether BAs can be used for biomarkers or therapeutics is yet to be determined and further studies are needed to elucidate the role of specific microbial species in the production of secondary BAs and their impact on ADRD pathophysiology.

### Tryptophan derivatives

Several *Lactobacillus* strains are known to grow on tryptophan to produce AhR ligands such as kynurenine and indoles [32]. The susceptibility to colitis of germ-free mice colonized with fecal microbiota from colitis mice was rescued by the supplementation of *Lactobacillus* strains (*L. murinus* CNCM I-5020, *L. reuteri* CNCM I-5022, and *L. taiwanensis* CNCM I-5019), which have the capacity to activate AhR by metabolizing tryptophan and producing AhR ligands [357]. Interestingly, another study showed the promoted growth of *L. reuteri* in *Ido1* knockout mice exposed to concentrations of tryptophan. Moreover, *L. reuteri* produced IAld, an indole derivative and a ligand for AhR. Several L. *reuteri* strains are used as probiotics that confer many beneficial effects in the host such as improved gut barrier function, lowered inflammation, and inhibition of oxidative stress [[358], [359], [360]]. A recent mechanistic study on *L. reuteri* has revealed its ability to convert kynurenine into kynurenic acid, a suggestive neuroprotective metabolite [182]. In contrast, another study showed that *L. reuteri* can unexpectedly promote experimental autoimmune encephalomyelitis by modulating CNS-targeted T cell responses [361]. Conflicting results on the ability of *L. reuteri* on the kynurenine pathway and its manipulation of disease pathology imply that the influence of *L. reuteri* may be strain-specific and require cautious application to patients. Although multiple probiotic strains possess the potential to produce kynurenine pathway metabolites such as kynurenine and kynurenic acid that are associated with improvement in neuroinflammation and brain functions, studies demonstrating their direct production of these metabolites linking with ADRD pathology are limited.

Gut microbes have been identified to promote TPH1 expression and serotonin production via the stimulatory effect of SCFA on enterochromaffin cells [34]. Administration of the probiotic species *Lactobacillus johnsonii* to rats showed decreased kynurenine production and confirmed the inhibition of IDO activity in the intestinal epithelial cells, which led to elevated levels of serotonin in ileum tissue and serum [362]. Several studies have suggested that probiotic strains of *Lactobacillus johnsonii* have a beneficial effect of on memory dysfunction. The potent probiotic strain *Lactobacillus johnsonii* BS15 was supplemented with ICR mice for 28 days. Probiotic-treated mice subjected to water-avoidance stress between 22 and 28 days displayed lower levels of antioxidant capacity and apoptosis in the hippocampus compared to controls [363]. The same group performed a similar study with *Lactobacillus johnsonii* BS15 treatment in mice exposed to restraint stress and showed increased proinflammatory cytokines expression, decreased expression levels of tight junction proteins, and altered hippocampus-related memory abilities [364]. Other studies have also shown the beneficial effect of *Lactobacillus johnsonii* strains on restoring gut permeability and preventing memory deficits in mice [[365], [366], [367]]. These findings suggest that some *Lactobacillus johnsonii* strains may regulate intestinal production of serotonin as well as intestinal integrity and immunity, which may ameliorate memory functions in the brain. In addition to the potential application of *Lactobacillus johnsonii* as probiotics, there are many other potent gut microbial modulations impacting alterations in serotonin levels in the brain that may have therapeutic effects in ADRD pathology, however, the data are preliminary to be implemented as therapeutics in patients and further intervention studies in animal models of AD or AD patients are needed [368].

IPA can cross the BBB and IPA may hypothetically prevent neurodegeneration via its reduction of pro-oxidant activity and electron leakage from mitochondria [41,42,279,369]. A clinical study on healthy elderly individuals supplemented with probiotic capsules including *Bifidobacterium bifidum* BGN4 and *Bifidobacterium longum* BORI showed an altered metabolic pathway related to tryptophan and elevated serum IPA levels compared to the placebo group [370]. Interestingly, the levels of IPA showed a positive association with the expression levels of BDNF in the probiotic group and a negative association with the placebo group [370]. Further *in vitro* treatment with IPA on microglial cells displayed lowered levels of TNF-α (proinflammatory) and enhanced expression levels of BDNF and nerve growth factor, suggesting that increases in IPA levels after probiotic supplementation may have neuroprotective effects [370]. Although IPA has been suggested to have positive impacts on intestinal barrier function and immunity via AhR or PXR mediations and also on neurons to inhibit acetylcholinesterase (AChE) activity, stress response through the hypothalamic-pituitary-adrenal (HPA) axis, and Aβ fibril formation [371], it is difficult to conclude that IPA is beneficial in ADRD pathology, given that several contrasting clinical reports found higher IPA concentrations in patients with AD, PD, and multiple sclerosis [[372], [373], [374]]. Further clinical and preclinical studies are needed to understand the role of IPA and other indoles in the pathophysiology of ADRD.

### TMA/TMAO

Since TMAO production results from gut microbial metabolism of nutrients found in the diet, it is plausible that a reduction in the consumption of TMAO precursors could prevent the deleterious effects of TMAO on disease. In a clinical study, it was found that patients with coronary artery disease who consume more choline- and l-carnitine-containing foods have higher TMAO levels in the blood, and have an increased abundance of gut bacteria associated with trimethylamine production [313]. In another study, vegetarians were found to have a lower TMAO production than omnivores [375]. These studies suggest that decreased consumption of foods containing TMAO precursors may prevent the disease-promoting effects of TMAO. However, the importance of consumption of TMAO precursors such as choline and carnitine must also be considered.

Modulation of the gut microbiota to promote bacterial strains without TMA-lyase activity shows promise in decreasing TMAO levels in circulation [376,377]. It has generally been found in preclinical models that *Lactobacillus* strains are effective probiotics in several diseases, including AD [[378], [379], [380]] and other diseases associated with increased TMAO levels [381]. One study found that probiotic administration of *L. amylovorus* and *L. plantarum*, both separately and together with *L. fermentum*, was effective in decreasing serum levels of TMA and TMAO in C57BL/6J mice [376,377]. Other studies have found that *L. rhamnosus* reduces TMAO levels in both humans and animals [377,382]. There is a need for further exploration of these strains in animal models of disease, including models of neurological diseases.

Gut microbes with choline TMA-lyase activity, encoded by the *cutC* gene, are required for the production of TMA from dietary nutrients, which is further metabolized to produce TMAO [45,49]. Studies have found that inhibitors of TMA-lyase are able to reduce TMAO production [311,383]. 3, 3-Dimethyl-1-butanol (DMB) is a structural analog of choline found to reduce plasma TMAO levels via inhibition of TMA-lyase activity as well as the reduction of TMA-producing bacteria in the gut [311,383]. DMB administration to APP/PS1 AD mice was found to reduce plasma TMAO levels, reduce expression of proinflammatory cytokines, reduce hippocampal inflammation, and ameliorate cognitive and pathological deterioration [58]. New TMA-lyase inhibitors iodomethylcholine (IMC) and fluoromethylcholine (FMC) have improved efficiency, reduced toxicity, and decreased plasma levels of TMAO [311,383]. IMC has also been found to have effects in the brain: it was found that mice have enriched TMAO at the olfactory bulb, and IMC administration to mice results in altered olfactory perception [384]. This study also found that IMC did not affect cognitive, anxiety-like, and depression-related behaviors, but had negative effects on some social behaviors, possibly due to impaired olfactory function [384]. Further studies on TMA-lyase inhibition effects in neurological diseases are needed.

Substantial evidence indicates that the gut microbiome is altered in ADRD. These differences in the composition of the gut microbiome are associated with alterations in the abundances of metabolites that gut microbes produce or modulate. Accumulating evidence supports the notion that at least some of these metabolites interact with the brain, impacting myriad pathways including neuronal signaling, immune function and inflammation, affecting progression of pathology.

A significant challenge in understanding the interaction between the gut microbiome and the host is that the association is bidirectional and not always causal to the host's physiology. There is also a myriad of factors to consider in the context of interpreting gut microbiota and brain interactions, including sex, age, genotype, interindividual variability, how the microbiome environment is affected over the life course [385] and may be influenced by lifestyle changes [386], and by host-microbe and microbe-microbe interactions [[387], [388], [389], [390]]. Despite multiple challenges in studying the impact of gut microbiome on host physiology, researchers have leveraged *in vivo* and *in vitro* models as well as human clinical studies including both observational and experimental (intervention) designs to understand mechanisms that gut microbes confer to host health and disease.

This review summarized available evidence on the role of gut microbes and microbial metabolites on ADRD pathology and cognitive and behavioral function in the context of ADRD. Although human clinical studies investigating the pathophysiology and therapeutics of specific microbes and their metabolites on ADRD are limited, mounting evidence from both preclinical and clinical studies suggests that changes in gut microbiota in the host are associated with ADRD pathology. This opens up the possibility of modulating gut microbes as potential targets for preventing the onset and/or progression of ADRD. Moreover, multi-omics approaches have expanded the opportunities to explore microbe-host interactions and to find target microbes or molecules that are unique and novel to the host pathophysiology. Linking gut microbiome composition and function leveraging microbial metagenomics, transcriptomics, and metabolomics together with the host genome, transcriptome, proteome, and metabolome as well as multiple biomarkers related to ADRD is required to understand the intricate and multifaceted interconnectome and to devise treatment strategies for early detection and to slow down the progression of ADRD. Finally, mechanistic research on therapeutics targeting gut microbiome is needed to enhance beneficial microbial metabolites and reduce deleterious ones associated with poor brain health. Research in this area is expected to inform the development of new therapeutics as well as precision, integrative, and preventive medicine for ADRD.

## Author contributions

J.W.K., V.V., and J.F.K. conceived and wrote the manuscript. J.W.K., V.V., and J.F.K. created figures and tables. J.W.K., V.V., J.F.K., T.K.U., F.E.R., and B.B.B. edited and reviewed the manuscript. All authors have read and agreed to the published version of the manuscript.

## Funding

This work was supported by the 10.13039/100000049National Institute on Aging Grant R01AG070973 (B.B.B., F.E.R., T.K.U.), 10.13039/100000049National Institute on Aging Grant R01AG083883 (T K.U., B.B.B., F.E.R.), Vilas Early-Career Investigator Award (T.K.U.), and 10.13039/100000049National Institute on Aging Grant P30AG062715 (B.B.B., T.K.U.).

## Declaration of competing interest

The authors declare that they have no known competing financial interests or personal relationships that could have appeared to influence the work reported in this paper.
